# High Plasma Levels of Soluble Talin-1 in Patients with Coronary Artery Disease

**DOI:** 10.1155/2020/2479830

**Published:** 2020-05-29

**Authors:** Masayuki Aoyama, Yoshimi Kishimoto, Emi Saita, Yukinori Ikegami, Reiko Ohmori, Masato Nakamura, Kazuo Kondo, Yukihiko Momiyama

**Affiliations:** ^1^Department of Cardiology, National Hospital Organization Tokyo Medical Center, Japan; ^2^Division of Cardiovascular Medicine, Toho University Ohashi Medical Center, Tokyo, Japan; ^3^Endowed Research Department “Food for Health”, Ochanomizu University, Tokyo, Japan; ^4^Faculty of Regional Design, Utsunomiya University, Tochigi, Japan; ^5^Institute of Life Innovation Studies, Toyo University, Gunma, Japan

## Abstract

**Aims:**

Talin-1 is a cytoskeletal protein that binds integrin, thereby leading to integrin activation and affecting focal adhesions. Recently, talin-1 expression was reported to be downregulated in human atherosclerotic plaques. However, blood levels of soluble talin-1 (sTalin-1) in patients with atherosclerotic disease, such as coronary artery disease (CAD), have not been elucidated.

**Methods:**

We measured plasma sTalin-1 levels in 349 patients undergoing elective coronary angiography. The severity of CAD was represented as the number of stenotic coronary vessels and segments.

**Results:**

Of the 349 study patients, CAD was found in 194 patients, of whom 88 had 1-vessel disease (1-VD), 60 had 2-vessel disease (2-VD), and 46 had 3-vessel disease (3-VD). Plasma sTalin-1 levels were higher in 194 patients with CAD than in 155 without CAD (CAD(-) group) (median 0.30 vs. 0.23 ng/mL, *P* < 0.005). A stepwise increase in sTalin-1 levels was found depending on the number of >50% stenotic coronary vessels: 0.23 in CAD(-), 0.29 in 1-VD, 0.30 in 2-VD, and 0.32 ng/mL in 3-VD group, respectively, (*P* < 0.05). High sTalin-1 level (>0.28 ng/mL) was found in 36% of CAD(-), 51% of 1-VD, 53% of 2-VD, and 59% of 3-VD group (*P* < 0.025). sTalin-1 levels also correlated with the number of >50% stenotic segments (*r* = 0.14, *P* < 0.02). The multivariate analysis revealed that sTalin-1 levels were independently associated with CAD. The odds ratio for CAD was 1.83 (95%CI = 1.14 − 2.93) for high sTalin-1 level (>0.28 ng/mL) (*P* < 0.02).

**Conclusions:**

Plasma sTalin-1 levels in patients with CAD were found to be high and to be associated with the presence and severity of CAD, suggesting a role of sTalin-1 in the progression of coronary atherosclerosis.

## 1. Introduction

Focal adhesions are key attachments between the cells and the extracellular matrix (ECM) and are important for maintaining cell integrity and intercellular communication. Integrins, which are transmembrane receptors composed of *α* and *β* subunits, are the major components of focal adhesions. Integrins play a pivotal role in the structural integrity of focal adhesions and in the cell-to-ECM adhesive interactions [1, 2].

Talin-1 is a dimeric cytoskeletal protein that binds integrin *β* subunits, thereby leading to integrin activation and affecting focal adhesions [2, 3]. Talin-1 is expressed in nearly all tissues, but mainly in the kidney, liver, spleen, lung, and vascular smooth muscle [1, 4, 5]. Talin-1 is recognized to be a potent integrin activator and to influence the integrin functions, which are associated with cell adhesions, migration, apoptosis, and cytoskeleton remodeling [1, 6]. Talin-1 also promotes cell proliferation by activating focal adhesion proteins and by influencing integrin adhesions with cell cycle progression [7].

Recently, von Essen et al. [8] reported that *TLN1* (a gene encoding talin-1) expression was downregulated in atherosclerotic plaques (68 samples from carotid, aortic or femoral arteries) compared with normal artery samples. They suggested that talin-1 downregulation causes the loosening of cell-ECM interactions, thereby leading to the injury and disintegration of vascular walls in atherosclerosis. Furthermore, talin-1 was shown to be downregulated in unstable carotid plaques compared with stable plaques [9]. Moreover, talin-1 was shown to be downregulated in the media of aortic samples from 10 patients with aortic dissection [10]. One proteomic study of 16 coronary arterial samples reported that 5 cytoskeleton proteins, including talin-1, were downregulated in atherosclerotic coronary media [11]. However, talin-1 levels in the blood of patients with atherosclerotic diseases, such as coronary artery disease (CAD), have not been elucidated yet. To elucidate blood talin-1 levels in patients with CAD, we measured plasma soluble talin-1 (sTalin-1) levels in 349 patients undergoing elective coronary angiography.

## 2. Methods

### 2.1. Study Patients

The data that support the findings of this study are available from the corresponding author on reasonable request. We investigated plasma sTalin-1 levels in 349 consecutive patients undergoing elective coronary angiography for suspected CAD at Tokyo Medical Center from June 2009 to September 2016. Any patients with acute coronary syndrome, defined as acute myocardial infarction and class III unstable angina at rest by Braunwald's classification [12], were excluded from this study. Patients with a history of heart failure or severe valvular heart disease and those with a history of percutaneous coronary intervention or cardiac surgery were also excluded. Moreover, patients with liver cirrhosis, renal failure, or inflammatory diseases were excluded. Since blood sTalin-1 levels were reported to be high in patients with colon cancer [13] and liver cancer [5], any patients with malignancy were excluded. Hypertension was defined as blood pressures of ≥140/90 mmHg or on drugs, and 202 (58%) patients were taking antihypertensive drugs. Hyperlipidemia was defined as an LDL-cholesterol level of >140 mg/dL or on drugs, and 129 (37%) patients were taking statins. Diabetes mellitus (DM) (a fasting plasma glucose [FPG] level of ≥126 mg/dL or on treatment) was present in 90 (26%) patients, and 122 (35%) were smokers (≥10 pack-years). Our study was approved by the institutional ethics committee of our hospital (R07-054/R15-056). After written informed consent was obtained, overnight-fasting blood samples were taken on the morning of the day when coronary angiography was performed.

### 2.2. Measurements of Plasma sTalin-1 and C-Reactive Protein (CRP) Levels

Blood samples were collected in EDTA-containing tubes and then were centrifuged at 2000 g for 15 minutes at 4°C. The plasma was frozen and stored at –80°C until analyzed. Plasma sTalin-1 levels were measured using an enzyme-linked immunosorbent assay (ELISA) with a commercially available kit (Human TLN (Talin) ELISA Kit, Elabscience, Houston, USA) at Ochanomizu University according to the manufacturer's instructions. According to the data supplied by the manufacturer, this kit shows no cross-reactivity with talin-2. The intra- and interassay coefficients of variation were <10% and <10%, respectively. Plasma high-sensitivity CRP (hsCRP) levels were also measured by a BNII nephelometer (Dade Behring, Tokyo, Japan).

### 2.3. Coronary Angiography

Angiograms were recorded on a cineangiogram system (Philips Electronics Japan, Tokyo, Japan). CAD was defined as at least one coronary artery having >50% luminal diameter stenosis on angiograms. The severity of CAD was represented as the numbers of >50% stenotic vessels and stenotic segments and the severity score of stenosis. The degree of coronary stenosis in each segment was scored from 0 to 4 points (0, ≤25%; 1, 26%-50%; 2, 51%-75%; 3, 76%-90%; 4, >90% stenosis), and then the severity score was defined as the sum of scores of all segments. Coronary artery segments were defined as 29 segments according to the Coronary Artery Surgery Study (CASS) classification. All angiograms were evaluated by a single cardiologist (Y.M.), who was blinded to the clinical and laboratory data.

### 2.4. Statistical Analysis

Differences between 2 groups were evaluated by unpaired *t*-test for parametric variables, by Mann-Whitney *U* test for nonparametric variables, and by chi-squared test for categorical variables. Differences among ≥3 groups were evaluated by an analysis of variance with Scheffe's test for parametric variables, by Kruskal-Wallis test for nonparametric variables, and by chi-squared test for categorical variables. Since the distributions of the measured sTalin-1 and hsCRP levels were considered to be highly skewed and to be nonparametric variables by Shapiro-Wilk test, their results were presented as the median value. Correlations between plasma sTalin-1 levels and lipid or hsCRP levels or the severity of CAD were evaluated by Spearman's rank correlation test. To determine the cut-off point of sTalin-1 levels for CAD, a receiver-operating characteristic (ROC) curve was created, and then the optimal cut-off point was determined to be 0.28 ng/mL as the point where the Youden index was maximum. The optimal cut-off point of age was also determined to be 70 years. Regarding the cut-off point of hsCRP levels, the previously reported cut-off point of 1.0 mg/L for CAD was used [14, 15]. The areas under ROC curves (AUC) were measured to compare the diagnostic abilities of sTalin-1 and hsCRP levels to predict CAD. A forward stepwise multiple logistic regression analysis was performed to determine the independent association between sTalin-1 levels and CAD. All statistical analyses were performed using the SPSS software package (IBM SPSS version 25, Japan). A *P* value of <0.05 was considered to be statistically significant. The results are presented as the mean ± SD or the median value.

## 3. Results

**Figure 1:**
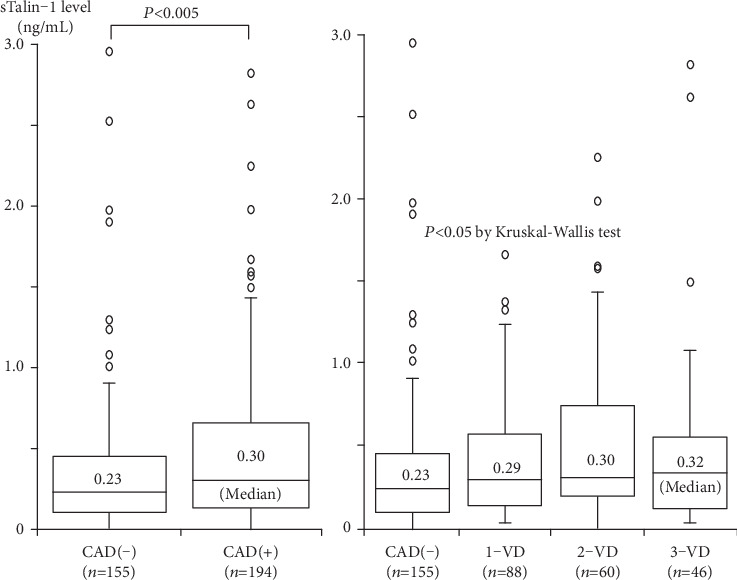
Plasma sTalin-1 levels and the presence of CAD or the number of stenotic coronary vessels. Plasma sTalin-1 levels were significantly higher in CAD than in CAD(-) (left). Moreover, sTalin-1 levels in 4 groups of CAD(-), 1-VD, 2-VD, and 3-VD were 0.23, 0.29, 0.30, and 0.32 ng/mL, respectively, and were highest in 3-VD (*P* < 0.05 by Kruskal-Wallis test) (right). The central line represents the median, and the box represents the 25th to 75th percentiles. The whiskers represent the lowest and highest value in the 25th percentile minus 1.5 IQR and 75th percentile plus 1.5 IQR, respectively.

Among the 349 study patients, CAD was present in 194 patients (56%) (1-vessel disease [1-VD], *n* = 88; 2-vessel disease [2-VD], *n* = 60; and 3-vessel disease [3-VD], *n* = 46). Compared with 155 patients without CAD (CAD(-) group), 194 patients with CAD (CAD group) were older and had a male predominance; higher prevalence of hypertension, hyperlipidemia, and DM; and lower HDL-cholesterol levels (Table 1). Plasma hsCRP levels were higher in CAD group than in CAD(-) group (median 0.58 vs. 0.42 mg/L, *P* < 0.001) (Table 1). A stepwise increase in hsCRP levels was also found depending on the number of >50% stenotic coronary vessels: 0.42 in CAD(-), 0.59 in 1-VD, 0.56 in 2-VD, and 0.70 ng/mL in 3-VD group, respectively (*P* < 0.002). Notably, plasma sTalin-1 levels were significantly higher in CAD than in CAD(-) (median 0.30 vs. 0.23 ng/mL, *P* < 0.005) (Figure 1). sTalin-1 levels also correlated with hsCRP (*rs* = 0.33, *P* < 0.001) and HDL-cholesterol (*rs* = −0.28, *P* < 0.001) levels but not with LDL-cholesterol levels. A stepwise increase in sTalin-1 levels was found depending on the number of >50% stenotic coronary vessels: 0.23 in CAD(-), 0.29 in 1-VD, 0.30 in 2-VD, and 0.32 ng/mL in 3-VD group, respectively (*P* < 0.05) (Figure 1). A high sTalin-1 level (>0.28 ng/mL) was present in 36% of CAD(-), 51% of 1-VD, 53% of 2-VD, and 59% of 3-VD group, respectively (*P* < 0.025) (Table 1). Furthermore, sTalin-1 levels significantly, but weakly, correlated with the number of >50% stenotic coronary segments and the severity score (*rs* = 0.14 and *rs* = 0.14, *P* < 0.02).

Regarding gender differences in clinical characteristics and sTalin-1 levels (Table 2), men were significantly younger and had higher prevalence of smoking and lower HDL-cholesterol levels than women. Furthermore, men more often had CAD than women. However, no significant difference was found in sTalin-1 levels between men and women.

**Figure 2:**
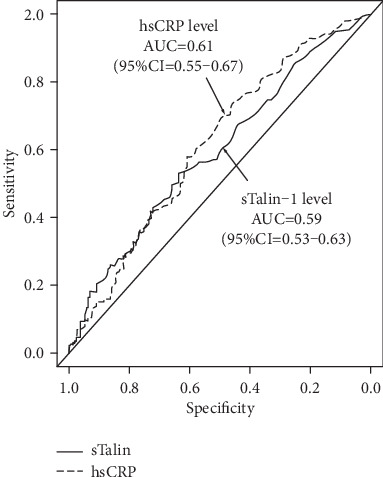
ROC curves of sTalin-1 and hsCRP levels for the diagnostic ability of CAD. Regarding the diagnostic abilities of sTalin-1 and hsCRP levels to predict CAD, the AUC for sTalin-1 levels was 0.59 (95%CI = 0.53–0.63), which did not significantly differ from the AUC for hsCRP levels (0.61; 95%CI = 0.55–0.67).

Regarding the diagnostic abilities of sTalin-1 and hsCRP levels to predict CAD, the AUC for sTalin-1 levels was 0.59 (95%CI = 0.53–0.63), which did not significantly differ from the AUC for hsCRP levels (0.61; 95%CI = 0.55–0.67) (Figure 2). To elucidate the independent association between plasma sTalin-1 levels and CAD, variables (age (>70 years), male gender, hypertension, hyperlipidemia, statin use, low HDL-cholesterol level (<40 mg/dL), DM, smoking, and high hsCRP (>1.0 mg/dL) and high sTalin-1 (>0.28 ng/mL) levels) were entered into a multiple logistic regression model. Plasma sTalin-1 levels were found to be a significant factor associated with CAD independent of atherosclerotic risk factors. The odds ratio for CAD was 1.83 (95%CI = 1.14 − 2.93) for the high sTalin level of >0.28 ng/mL (*P* < 0.02) (Table 3).

## 4. Discussion

In the present study, plasma sTalin-1 levels were significantly higher in patients with CAD than in those without CAD, and they positively correlated with the severity of CAD, defined as the numbers of stenotic vessels and segments and the severity score. High plasma sTalin-1 levels were a significant factor associated with CAD independent of atherosclerotic risk factors.

Talin-1 is an integrin activator that affects cell adhesions, migration, and apoptosis [1, 6]. Talin-1 is also recognized to regulate platelet and leukocyte integrin functions [16]. Talin-1 was reported to increase platelet adhesion and aggregation, while Talin-1 deficiency caused severe hemostatic defects and resistance to arterial thrombosis [17]. Moreover, Talin-1 has been shown to regulate *α*4*β*1 integrin in lymphocytes, interacting with endothelial vascular cell adhesion molecule-1 (VCAM-1) on blood vessels [6]. In inflammation, neutrophils and other leukocytes roll along microvascular endothelium before arresting and transmigrating into inflamed tissues. Using ex vivo and in vivo assays, Talin-1 was shown to play a major role in neutrophil slow rolling and arrest [18]. These findings thus suggest that talin-1 may affect the progression of atherosclerosis.

Regarding the association between talin-1 and atherosclerosis, *TLN1* expression was demonstrated to be downregulated in atherosclerotic plaques [8]. One proteomic study also showed the downregulation of talin-1 in atherosclerotic coronary media [11]. These findings thus suggest that talin-1 would be downregulated in atherosclerotic plaques. Furthermore, talin-1 was reported to be downregulated in unstable carotid plaques compared with stable plaques [9]. The overexpression of microRNA-330-5p was also demonstrated in unstable plaques, which significantly downregulated talin-1 [9]. In contrast, high *TLN1* expression along with low microRNA-330-5p expression was reported in hepatocellular cancer tissues [19]. These suggest that microRNA-330-5p may play a role in talin-1 downregulation in atherosclerotic plaques. However, no study has reported blood sTalin-1 levels of patients with CAD.

Interestingly, we demonstrated that plasma sTalin-1 levels in patients with CAD were high. Previously, serum sTalin-1 levels were reported to be high in patients with colon cancer [13] and in those with hepatocellular cancer [5]. Recently, Muto et al. [20] also reported that serum sTalin-1 levels were higher in 40 patients with multiple sclerosis (MS) than in 43 controls and were higher in the acute phase than in the remission phase. Another study by Muto et al. [21] also showed that serum antibodies against talin-1 were higher in 39 patients with MS than in 43 controls. MS is an autoimmune, demyelinating disease, and T cells are believed to play a pivotal role in its pathogenesis [20]. They suggested that talin-1 may be released into blood as extracellular vesicles from damaged or stressed cells in such patients. Furthermore, plasma sTalin-1 levels were reported to be higher in 50 patients with rheumatoid arthritis, which is a chronic inflammatory disease, than in 20 controls [22]. These findings therefore suggest that patients with chronic inflammatory diseases may have high blood levels of sTalin-1. One proteomic analysis of plasma samples demonstrated the upregulation of talin-1 in 45 patients with lacunar infarction, especially in those with adverse outcomes [23]. However, another proteomic analysis reported no significant difference in plasma sTalin-1 levels between 30 subjects with coronary artery calcium and 30 without it detected by computed tomography [24]. Notably, we found that plasma sTalin-1 levels in patients with CAD were high and that they positively correlated with the severity of CAD. High sTalin-1 levels were a significant factor associated with CAD. Our results thus suggest that patients with CAD had high plasma levels of sTalin-1, which may play a role in the progression of coronary atherosclerosis. Moreover, sTalin-1 levels also correlated with hsCRP levels. Since inflammation has been recognized to play an important role in atherosclerosis [25], high sTalin-1 levels may reflect inflammation in coronary atherosclerosis.

Because talin-1 expression was reported to be downregulated in atherosclerotic plaques of carotid, aortic, or femoral arteries [8] and in atherosclerotic coronary media [11], it can be speculated that talin-1 expression in coronary atherosclerotic lesions of patients with CAD would be downregulated. However, we found that plasma sTalin-1 levels in patients with CAD were high and that sTalin-1 levels correlated with the severity of coronary atherosclerosis as well as plasma hsCRP levels. Therefore, high sTalin-1 levels in patients with CAD may reflect inflammation in coronary atherosclerosis or represent an adaptive response to low talin-1 expression in coronary atherosclerotic plaques. As in patients with CAD, blood sTalin-1 levels were reported to be high in patients with cancer [5, 13], MS [20], rheumatoid arthritis [22], and lacunar infarction [23]. However, the main sources of blood sTalin-1 in these patients and the mechanism of how intracellular talin-1 is released into the blood have not been elucidated yet. Inflammation activates macrophages by ATP stimulation and then leads to the activation of calpains, which are nonlysosomal cysteine proteases [26]. Calpains are involved in inflammatory processes and activated calpains damage cells by degrading intracellular proteins, including talin-1 [27, 28]. Calpains exacerbate inflammation and atherosclerosis in athrosclerotic models [29]. Most talin-1 is present in cytosol and translocates to cell membrane in response to the activation of Rap1A GTPase, a key regulator of integrin activity [30, 31]. Calpains were shown to play a major role in exosomal secretion of intracellular or membrane proteins, including talin-1. Talin-1 was demonstrated to be exosomally secreted from human macrophages by ATP stimulation [26]. However, further studies are needed to elucidate the major source of sTalin-1 in patients with CAD and the role of high blood levels of sTalin-1 in the progression of coronary atherosclerosis. As shown in Figure 1, there was a substantial overlap in plasma sTalin-1 levels between patients with and without CAD, and the correlation between sTalin-1 levels and the severity of CAD, defined as the number of stenotic segments and the severity score, was significant but weak (*r* = 0.14). Thus, plasma sTalin-1 levels in patients with CAD may reflect not only the severity of coronary atherosclerosis but also atherosclerosis in other vascular beds. Moreover, because talin-1 may be released into the blood as extracellular vesicles from damaged or stressed cells or inflammatory cells [20, 26], sTalin-1 levels may also reflect inflammation or stress conditions in nonvascular tissues.

Some gender differences in clinical characteristics of patients with CAD have been reported [32, 33]. Men with CAD are known to have higher prevalence of smoking and lower HDL-cholesterol levels than in women [32]. The prevalence of obstructive CAD is more common in men with CAD than in such women [33]. Our study also showed that men were significantly younger and had higher prevalence of smoking and lower HDL-cholesterol levels than women. Moreover, men more often had CAD. However, there was no significant difference in sTalin-1 levels between men and women.

Our study has several limitations. First, talin-1 (2541 amino acids) are comprised of an *N*-terminal head (1-400), a linker (401-481), and a rod (482-2541), and talin-1 is cleavaged into several fragments by calpain protease [3, 34]. Calpain cleavage sites are present at a linker and near the C-terminal of a rod. However, the ELISA kit used in our study recognizes a 700-860 amino acids sequence of human talin-1. Therefore, this kit cannot detect any fragment that does not contain that sequence. This may have affected our results. Moreover, this kit has no cross-reactivity with talin-2, but the intra- and interassay coefficients of variation were <10% and <10%, respectively. However, sTalin-1 levels in patients with CAD were 30% higher than in those without CAD (0.30 vs. 0.23 ng/mL). Second, in our study, angiography was used to evaluated coronary atherosclerosis. Coronary angiography cannot visualize plaques and only shows lumen characteristics. However, intravascular ultrasound (IVUS), which can visualize coronary plaques, was not always performed in our study patients. Third, no patient with acute coronary syndrome was included in our study. A further study in patients with acute coronary syndrome, such as unstable angina, is needed to elucidate the potential role of sTalin-1 in this syndrome. Fourth, our study did not analyze any variants of *TLN1* gene. Since some *TLN1* gene variants were reported to be associated with coronary artery dissection [35], such variants may have affected plasma sTalin-1 levels in our patients with CAD. Fifth, our study was cross-sectional in nature and was unable to establish causality, since it only depicted some associations and proposed some hypotheses. Sixth, we had no healthy controls. We investigated 349 patients undergoing angiography, who were divided into two groups with and without CAD. Some patients without CAD had mild, but not significant, stenosis. These may have confounded the results. Moreover, as in our previous study [36], our study was in Japanese patients undergoing coronary angiography, who are generally considered to be a highly selected population at high-risk for CAD. Our results may not be applicable to the general or other ethnic populations. Finally, the number of our study patients is not so large. However, the sample size of our study (349 patients) was found to be enough to show a 1.83-fold higher risk of CAD in patients with high sTalin-1 level (>0.28 ng/mL) with a statistical power of 80% and an *α* value of 0.05, because 212 patients were estimated as the adequate size with the prevalence of CAD (54%).

In conclusion, plasma sTalin-1 levels in patients with CAD were found to be high and to be associated with the presence and severity of CAD. Our results thus suggest that high sTalin-1 levels in patients with CAD may play a role in the progression of CAD. Moreover, since plasma sTalin-1 levels correlated with hsCRP levels, sTalin-1 levels may reflect inflammation in coronary atherosclerosis. However, further studies are needed to elucidate the main sources and role of sTalin-1 in blood in the progression of coronary atherosclerosis.

## Figures and Tables

**Table 1:** Clinical characteristics and plasma sTalin-1 levels of patients with and without CAD.

	CAD(-) (*n* = 155)	*P* value	CAD (*n* = 194)	1-VD (*n* = 88)	2-VD (*n* = 60)	3-VD (*n* = 46)	*P* value among 4 groups
		CAD(-) vs. CAD					
Age (years)	65 ± 12	<0.001	69 ± 10	68 ± 11	69 ± 10	72 ± 8	<0.001
Gender (male)	95 (61%)	<0.01	145 (75%)	64 (73%)	43 (72%)	38 (83%)	<0.05
BMI (kg/m^2^)	23.8 ± 3.7	NS	24.0 ± 3.8	24.6 ± 4.3	23.8 ± 3.0	23.1 ± 3.6	NS
Hypertension	93 (60%)	<0.001	151 (78%)	66 (75%)	46 (77%)	39 (85%)	<0.005
SBP (mmHg)	129 ± 21	<0.05	134 ± 19	133 ± 17	139 ± 21	130 ± 17	<0.02
Diabetes mellitus	24 (15%)	<0.001	66 (34%)	25 (28%)	23 (38%)	18 (39%)	<0.001
HbA1c (%)	6.0 ± 0.8	<0.001	6.3 ± 0.9	6.2 ± 0.8	6.4 ± 1.1	6.3 ± 0.9	<0.005
Smoking	45 (29%)	NS	77 (40%)	38 (43%)	25 (42%)	14 (30%)	NS
Hyperlipidemia	67 (43%)	<0.01	113 (58%)	51 (58%)	35 (58%)	27 (59%)	NS
Statin	42 (27%)	<0.001	87 (45%)	39 (44%)	26 (43%)	22 (48%)	<0.01
LDL-C (mg/dL)	114 ± 28	NS	115 ± 32	113 ± 33	116 ± 32	118 ± 29	NS
HDL-C (mg/dL)	59 ± 15	<0.001	52 ± 14	55 ± 14	51 ± 12	48 ± 13	<0.001
<40 mg/dL	14 (9%)	<0.05	34 (18%)	11 (13%)	11 (18%)	12 (26%)	<0.025
hsCRP (mg/L)	0.42 [0.21, 0.88]	<0.001	0.58 [0.33, 1.40]	0.59 [0.31, 1.35]	0.56 [0.30, 1.14]	0.70 [0.42, 2.03]	<0.002
>1.0 mg/L	35 (23%)	NS	63 (32%)	26 (30%)	19 (32%)	18 (39%)	NS
sTalin-1 levels (ng/mL)	0.23 [0.09, 0.44]	<0.005	0.30 [0.13, 0.66]	0.29 [0.12, 0.56]	0.30 [0.18, 0.72]	0.32 [0.11, 0.60]	<0.05
>0.28 ng/mL	56 (36%)	<0.005	104 (54%)	45 (51%)	32 (53%)	27 (59%)	<0.025

Data represent the mean ± SD or the number (%) of patients, with the exception of hsCRP and sTalin-1 levels which are presented as the median value and interquartile range. BMI indicates body mass index; SBP: systolic blood pressure; LDL-C: low-density lipoprotein cholesterol; and HDL-C: high-density lipoprotein cholesterol.

**Table 2:** Gender differences in clinical characteristics and plasma sTalin-1 levels.

	Male (*n* = 240)	*P* value	Female (*n* = 109)
Age (years)	66 ± 11	<0.005	69 ± 10
BMI (kg/m^2^)	24.3 ± 3.7	<0.01	23.1 ± 3.7
Hypertension	173 (72%)	NS	71 (65%)
SBP (mmHg)	132 ± 18	NS	132 ± 22
Diabetes mellitus	72 (30%)	<0.025	18 (17%)
HbA1c (%)	6.2 ± 0.9	NS	6.0 ± 0.6
Smoking	105 (44%)	<0.001	17 (16%)
Hyperlipidemia	104 (43%)	<0.001	76 (70%)
Statin	75 (31%)	<0.005	54 (50%)
LDL-C (mg/dL)	112 ± 28	NS	119 ± 34
HDL-C (mg/dL)	52 ± 14	<0.001	62 ± 13
hsCRP (mg/L)	0.56 [0.30, 1.21]	NS	0.39 [0.26, 1.09]
sTalin-1 levels (ng/mL)	0.28 [0.12, 0.53]	NS	0.24 [0.12, 0.53]
CAD	145 (60%)	<0.01	49 (45%)

Data represent the mean ± SD or the number (%) of patients, with the exception of hsCRP and sTalin-1 levels which are presented as the median value and interquartile range.

**Table 3:** Factors associated with CAD (multiple logistic regression analysis of the 349 study patients).

	Odds ratio	(95% CI)	*P* value
Age (>70 years)	2.26	(1.40-3.63)	<0.002
Male gender	2.01	(1.19-3.40)	<0.01
Statin use	1.90	(1.13-3.21)	<0.02
DM	2.09	(1.18-3.70)	<0.02
High sTalin-1 level (>0.28 ng/mL)	1.83	(1.14-2.93)	<0.02

The dependent variable was the presence of CAD. The analysis included age (>70 years), male gender, hypertension, hyperlipidemia, low HDL-cholesterol (<40 mg/dL), statin use, DM, smoking, and high hsCRP (>1.0 mg/L) and high sTalin-1 (>0.28 ng/mL) levels.

## Data Availability

We described in Methods (page 4) as follows: the data that support the findings of this study are available from the corresponding author on reasonable request.
